# Prevalence of comorbidities and secondary health conditions among the Finnish population with spinal cord injury

**DOI:** 10.1038/s41393-021-00704-7

**Published:** 2021-09-11

**Authors:** Susanna Tallqvist, Anna-Maija Kauppila, Aki Vainionpää, Eerika Koskinen, Paula Bergman, Heidi Anttila, Harri Hämäläinen, Anni Täckman, Mauri Kallinen, Jari Arokoski, Sinikka Hiekkala

**Affiliations:** 1 University of Helsinki, Faculty of Medicine, Helsinki, Finland; 2 Oulu University Hospital, Department of Medical Rehabilitation/Spinal Cord Injury Outpatient Clinic, Oulu, Finland; 3 Seinäjoki Central Hospital, Department of Rehabilitation, Seinäjoki, Finland; 4 Tampere University Hospital, Department of Neurosciences and Rehabilitation, Tampere, Finland; 5 Biostatistics Unit, Department of Public Health, University of Helsinki and Helsinki University Hospital, Helsinki, Finland; 6 Finnish Institute for Health and Welfare (THL), Public Health and Welfare Department, Knowledge Management and Co-creation Unit, Helsinki, Finland; 7 Helsinki University Hospital, Department of Internal Medicine and Rehabilitation/Spinal Cord Injury Outpatient Clinic, Helsinki, Finland; 8 The Finnish Association of Spinal Cord Injured Akson, Helsinki, Finland; 9 Department of Rehabilitation Medicine, Central Finland Health Care District, Central Finland Central Hospital, Jyväskylä, Finland; 10 Center for Life Course Health Research, University of Oulu, Oulu, Finland; 11 The Finnish Association of People with Physical Disabilities, Helsinki, Finland; 12 Validia Rehabilitation, Helsinki, Finland

**Keywords:** Rehabilitation, Spinal cord diseases

## Abstract

**Study design:**

A cross-sectional study.

**Objectives:**

To explore the prevalence of comorbidities, secondary health conditions (SHCs), and multimorbidity in the Finnish population with spinal cord injury (SCI).

**Setting:**

The data were collected from the Finnish Spinal Cord Injury Study (FinSCI). Participants were identified from three SCI outpatient clinics responsible for the lifelong follow-up of persons with SCI in Finland, (*n* = 884 participants, response rate; 50%).

**Methods:**

The FinSCI-questionnaire included a question from the National Study of Health, Well-being, and Service (FinSote) for screening 12 comorbidities. The reference data of the general population for that question were received from the Finnish Institute for Health and Welfare. The Spinal Cord Injury Secondary Condition Scale (SCI-SCS) was used to screen 16 SHCs. The data were analysed with univariate testing and multivariable negative binomial regression modelling.

**Results:**

The most common comorbidities were high blood pressure/hypertension (38%), back problems (28%), and high cholesterol (22%). The most common SHCs were joint and muscle pain (81%), muscle spasms (74%), chronic pain (71%), and bowel problems (71%). The prevalence of comorbidities was highest among persons aged ≥76 years (mean; 2.0; scale range; 0–12). The prevalence of SHCs was highest in the severity of SCI group C1–4 AIS A, B, and C (mean; 8.9; scale range; 0–16).

**Conclusions:**

Further research on geriatrics in SCI, non-traumatic SCI, and knowledge of the needs of persons with cervical lesion AIS A, B, or C is required, due to the fact that the prevalence of multimorbidity is high in these groups.

## Introduction

Persons with spinal cord injury (SCI) can experience several comorbidities and secondary health conditions (SHCs) after SCI. The word “comorbidity” refers to the presence of an additional disease in relation to an index disease in one person [1], whereas a “secondary health condition” is a health problem that is either a direct result of the impairment (like SCI) or an indirect factor that is related to the impairment [2]. Arthritis, hypertension, hyperlipidaemia, obesity, depression, diabetes, and alcoholism have been reported as some of the most common comorbidities among persons with SCI [3, 4]. Similarly, spasticity, chronic pain, sexual dysfunction, bowel and bladder dysfunction, urinary tract infections, sleep problems, and contractures are often experienced as health problems following SCI [5–8]. “Comorbidity” and “SHC” can be interpreted as synonyms. Terms are differentiated in order to clarify the interpretation of the results, and with the word “comorbidity” we refer to a disease that is treated or diagnosed by a physician. Persons with SCI can suffer from ≥2 comorbidities and/or SHCs simultaneously, which is known as a multimorbidity [1]. Based on a recent study, age and lesion characteristics had effects on multimorbidity in the analyses of 15 different SHCs [5].

The presence of comorbidities and/or SHCs is a burden to one’s health [1, 2], and places demands on health services [1]. As part of the Finnish Spinal Cord Injury Study (FinSCI) [9], the purpose of this article was to describe the prevalence of comorbidities and SHCs, as well as the prevalence of multimorbidity in the Finnish population with SCI. This article also compared the prevalence of these comorbidities and SHCs in relation to age and gender (later referred to as general characteristics), as well as the severity of SCI, aetiology, and time since injury (later referred to as lesion characteristics). The results of this article are focused on the general characteristics and severity of SCI. The prevalence of comorbidities and SHCs has not yet been studied among the Finnish population with SCI. Furthermore, it is important to understand the status of these health problems because Finland is reforming its health care system and the number of persons with SCI is increasing [10, 11].

## Methods

### Design

FinSCI is a collaborative study among The Finnish Association of People with Physical Disabilities, The Finnish Association of Spinal Cord Injured Akson, The Finnish Institute for Health and Welfare (THL), and the SCI outpatient clinics of three university hospitals (Oulu, Tampere, and Helsinki). The catchment population and locations of these hospitals are presented in Supplement (Figure A). The purpose of the FinSCI is to identify the factors that are related to the health and functioning of persons with SCI, their challenges with environmental factors of accessibility, and how such factors are interconnected [9].

### Sample

Study participants were identified from the registers of the SCI outpatient clinics from the university hospitals in Oulu, Tampere, and Helsinki, where their clinical data were also collected. The inclusion criteria were as follows: age of at least 16 years; non-traumatic SCI (NTSCI) or traumatic SCI (TSCI); and AIS classification of A, B, C, or D. International Standards for Neurological Classification of SCI (ISNCSCI) was used to determine The American Spinal Injury Association Impairment Scale (AIS) [12]. Patients with AIS E, living in an institute, or having a congenital SCI, progressive or neurodegenerative disease, multiple sclerosis, amyotrophic lateral sclerosis, or Guillain-Barre syndrome were excluded. The detailed protocol, the precise patient selection process, the ethical considerations, and the content of the formulated questionnaire have been presented in a separate publication [9]. The questionnaire was sent home to respondents in February 2019, and the answers were collected until the end of July 2019.

### Outcome measures

A questionnaire from the National Survey of Health, Well-being, and Service (FinSote) was applied to screen 12 comorbidities [13] and the Spinal Cord Injury Secondary Condition Scale (SCI-SCS) was used to screen 16 SHCs [14]. The prevalence of multimorbidity was analysed separately for comorbidities and SHCs. FinSote and SCI-SCS are self-reported outcome measures in which the data are collected from patients/persons who are living with their health condition [15]. SCI-SCS is also used in the International Spinal Cord Injury Survey, InSCI [16], in which FinSCI is not involved due to differences in study schedules and partly different self-reported outcome measures. The FinSCI study uses the International Classification of Functioning, Disability, and Health (ICF) [17] as a theoretical framework, and this article focuses on the analyses of body functions categories and health conditions (Supplementary Table A).

FinSote is a survey that is performed by the THL. The data are collected in 10,000 randomly selected individuals aged 20 years or older annually, and in 60,000 individuals every four years [13]. The relevance of the statistical analyses and the description of the utilised methods have been comprehensively reported by the THL [18]. This article analysed the prevalence of 12 different comorbidities that were diagnosed or treated by a physician during the last 12 months. The THL provides open access data concerning the four most common comorbidities: high blood pressure/hypertension, spondylosis, sciatica or other spinal disease (later referred to as back problems), high cholesterol, and coronary heart disease (CHD) or angina pectoris. Comparisons between the population with SCI and the reference group were conducted among these four illnesses. Information on the presence of CHD was only requested from participants who were aged 54 or older in the FinSote survey [18]. This study used the FinSote 2018 data as a reference, and the age groups were reclassified for this study by the THL.

The SCI-SCS is a self-reported health instrument scale that is used by persons with SCI, in which respondents are asked to rate their health over the last 3 months. The measure comprises 16 symptoms or conditions, 2 of which (diabetes mellitus and heterotopic ossification) should also be diagnosed by physicians. SHCs are evaluated on a scale from 0 to 3 (0 = not experienced in the last 3 months or is an insignificant problem; 1 = mild or infrequent problem; 2 = moderate or occasional problem; and 3 = significant or chronic problem), with a maximum score of 48. The higher the score, the more health problems the respondent experiences [14, 19]. The SCI-SCS has demonstrated reliability and validity, and it is included in the Spinal Cord Injury Research Evidence database as a recommended health measure to be used for persons with SCI [20].

### Statistical analyses

Duplicate responses were checked, and the second response was omitted. There were few missing values in the data (0.4–4.5% per question), in which case no imputation was performed. The data concerning the national reference group were received from the THL, where the missing values were corrected by using the inverse probability weighting method (IPW). The IPW method calculates a weighting factor for each respondent by taking into account the following variables: age, gender, marital status, level of education, province of residence, and its population density.

Descriptive statistics for the general and lesion characteristics, and results are presented as means (and standard deviations), medians (and interquartile ranges), and frequencies (and percentages), as appropriate. To assess the generalisability of the results, an analysis between participants and non-participants was performed by chi-square tests. Chi-square tests were also used to analyse the between group comparisons among the general and lesion characteristics. *P*-values for the pairwise comparisons were adjusted by the use of Bonferroni correction. The Z-test from Epitools—Epidemiological Calculators were used to compare the proportions of the comorbidities between the reference population and the population with SCI [21]. Multimorbidity was evaluated as the number of reported comorbidities (scale range 0–12) and SHCs (scale range 0–16). Two separate negative binomial regression models were used to examine the associations between general and lesion characteristics, and comorbidities and SCHs. Negative binomial regression modelling was used to estimate unadjusted and adjusted incidence rate ratios for the general and lesion characteristics, taking into account the overdispersion in the multimorbidity count data. Additionally, 95% confidence intervals (CIs) were used, and *p*-values of <0.05 were considered to be statistically significant. Statistical analyses were performed using SPSS version 25.

## Results

### Characteristics of participants and non-respondents

The response rate in the survey was 50%, and the final number of participants was 884. Participants and non-respondents were categorised according to the recommendations of the International Spinal Cord Injury Core Data Set (Table 1) [12]. The youngest participant was 20 years old, which is why the first age group is 20–30 years. In the analyses between participants (*n* = 884) and non-respondents (*n* = 888), there was a statistically significant difference between genders and age groups; younger persons aged 20–45 years answered less frequently, and persons 61–75 years of age, as well as females, actively participated. Two participants used a ventilator and were included in group C1–4 AIS A, B, and C based on the recommendations of the International Spinal Cord Injury Core Data Set [12]. The shortest time since an injury was 11 months and this value was rounded up to one year. There was one transgender participant who was grouped by the gender according to the hospital records. An internal analysis of the participants indicated that group AIS D in all injury levels (later referred to as group AIS D) differed from the other severity of SCI groups (Supplementary Table B).

**Table 1:** Eligible population of the Finnish Spinal Cord Injury study divided to participants (N884) and non-respondents (N888).

	Participants N884	Non-respondents N888	*p* value
	*n* (%)	*n* (%)	
Gender			<0.01
Male	577 (65%)	633 (71%)	
Female	307 (35%)	253 (29%)	
Aetiology			0.10
Traumatic	492 (56%)	527 (59%)	
Non-traumatic	392 (44%)	361 (41%)	
Severity of SCI			0.21
C1-4 AIS A, B, and C	95 (12%)	107 (11%)	
C5-8 AIS A, B, and C	55 (6%)	62 (7%)	
T1-S5 AIS A, B, and C	184 (21%)	209 (24%)	
AIS D at any injury level	550 (62%)	510 (57%)	
Age, years	min 20, max 90, mean 61, SD 14	min 17, max 93, mean 54, SD 17	<0.01
	median 63 IQR 53–71	median 55 IQR 40–68	
20–30	34 (4%)	96 (11%)	
31–45	108 (12%)	204 (23%)	
46–60	238 (27%)	243 (27 %)	
61–75	386 (44%)	243 (27%)	
≥ 76	118 (13%)	102 (12%)	
Time since injury, years	min 1, max 67, mean 11, SD 11	min 1, max 66, mean 10, SD 10	0.52
	median 7 IQR 4–14	median 6 IQR 4–14	
1–5 years	353 (40%)	379 (43%)	
6–10 years	227 (26%)	222 (25%)	
11–15 years	128 (14%)	111 (12%)	
≥ 16 years	176 (20%)	176 (20%)	

### Comorbidities

**Fig. 1:**
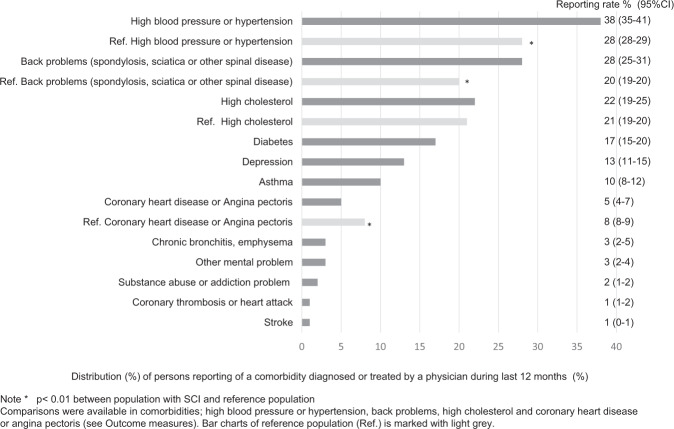
Prevalence of comorbidities (percentage with 95% confidence interval) diagnosed or treated by a physician during the last 12 months in the Finnish Spinal Cord Injury study (884 participants) and the reference population (Ref.).

There were 287 persons (33%) with SCI who answered that they had none of the 12 comorbidities. The two most common comorbidities diagnosed or treated in more than 25% of the population with SCI in Finland during the last 12 months were high blood pressure/hypertension and back problems (Fig. 1). The population with SCI had more of these problems than the reference population (Table 2a). The reference data were available only for the four most common comorbidities (high blood pressure/hypertension, back problems, high cholesterol, and CHD or angina pectoris). High cholesterol was the third most common comorbidity; in both populations it manifested in the same manner, but was more common among the reference population aged 61–75 years. In the subgroup analyses, CHD or angina pectoris was higher among the male reference group compared to males with SCI, and no difference between female reference and SCI groups (Table 2a).

**Table 2:** a Relative frequency (percentage with 95% confidence interval) of four most common comorbidity in the Finnish Spinal Cord Injury study (884 participants) and reference population in Finland, stratified by gender and age groups. b Relative frequency (percentage with 95% confidence interval) of comorbidities diagnosed or treated by a physician in the Finnish Spinal Cord Injury study (884 participants), stratified by gender, age groups, and severity of SCI.

		Coronary heart disease or Angina pectoris	High cholesterol	High blood pressure or hypertension	Back problems (spondylosis, sciatica, or other spinal disease)
Gender					
SCI Male	% (95% CI)	5 (3–6)	21 (18–25)	37 (33–41)	26 (22–29)
Ref. Male	% (95% CI)	10 (9–11)	21 (21–22)	30 (29–31)	19 (18–20)
	*p*	<0.01	1	<0.01	<0.01
SCI Female	% (95% CI)	6 (3–9)	23 (18–28)	41 (35–46)	33 (27–38)
Ref. Female	% (95% CI)	7 (6–7)	20 (19–20)	27 (26–27)	20 (20–21)
	*p*	0.50	0.20	<0.01	<0.01
Age groups					
SCI 20–30	% (95% CI)	0 (0–0)	0 (0–0)	0 (0–0)	13 (0–25)
Ref. 20–30	% (95% CI)		3 (2–3)	3 (3–4)	6 (6–7)
	*p*		0.37	0.30	0.13
SCI 31–45	% (95% CI)	0 (0–0)	8 (2–13)	10 (4–16)	15 (8–22)
Ref 31–45	% (95% CI)		8 (8–9)	8 (7–8)	15 (15–17)
	*p*		0.94	0.34	0.85
SCI 46-60	% (95% CI)	1 (0–3)	19 (13–24)	35 (29–42)	27 (21–33)
Ref 46–60	% (95% CI)	2 (2–3)	21 (21–23)	30 (29–31)	21 (20–22)
	*p*	0.22	0.35	0.10	<0.01
SCI 61–75	% (95% CI)	7 (4–9)	27 (23–32)	45 (40–50)	28 (24–33)
Ref 61–75	% (95% CI)	6 (6–7)	36 (34–37)	47 (45–48)	25 (24–26)
	*p*	0.53	<0.01	0.44	0.19
SCI ≥ 76	% (95% CI)	14 (7–20)	29 (21–37)	56 (47–65)	45 (36–54)
Ref. ≥ 76	% (95% CI)	18 (17–20)	36 (34–38)	62 (60–63)	32 (30–34)
	*p*	0.26	0.12	0.18	<0.01

Comorbidity	Gender			Age groups					Severity of SCI					
	Male	Female		20–30	31–45	46–60	61–75	≥76		C1-4 AIS A, B, and C	C5-8 AIS A, B, and C	T1-S5 AIS A, B, and C	AIS D at any injury level	
	% (95% CI)	% (95% CI)	*p*	% (95% CI)	% (95% CI)	% (95% CI)	% (95% CI)	% (95% CI)	*p*	% (95% CI)	% (95% CI)	% (95% CI)	% (95% CI)	*p*
High blood pressure or hypertension	37 (33–41)	41 (35–46)	0.30	0 (0–0)	10 (4–16)	35 (29–42)	45 (40–50)	56 (47–65)	<0.01	25 (16–34)	11 (2–19)	38 (31–45)	43 (39–47)	<0.01
Back problems (spondylosis, sciatica or other spinal disease)	26 (22–29)	33 (27–38)	0.03	13 (0–25)	15 (8–22)	27 (21–33)	28 (24–33)	45 (36–54)	<0.01	16 (9–24)	15 (5–24)	19 (13–25)	35 (31–39)	<0.01
High cholesterol	21 (18–25)	23 (18–28)	0.52	0 (0–0)	8 (2–13)	19 (13–24)	27 (23–32)	29 (21–37)	<0.01	16 (9–24)	9 (1–17)	25 (19–32)	23 (19–26)	0.04
Diabetes	16 (13–19)	19 (15–24)	0.28	3 (0–10)	4 (0–7)	13 (9–18)	20 (16–24)	31 (22–39)	<0.01	17 (9–25)	20 (9–31)	13 (8–18)	18 (15–22)	0.32
Depression	11 (9–14)	16 (12–20)	0.07	13 (0–25)	14 (7–21)	19 (14–25)	9 (6–12)	13 (7–19)	0.01	12 (5–19)	13 (4–22)	13 (8–18)	13 (10–16)	0.99
Asthma	7 (5–9)	15 (11–19)	<0.01	3 (0–10)	7 (2–11)	8 (5–12)	10 (87–14)	15 (9–22)	0.11	12 (5–19)	11 (2–19)	7 (3–11)	10 (8–13)	0.56
Coronary heart disease or Angina pectoris	5 (3–6)	6 (3–9)	0.39	0 (0–0)	0 (0–0)	1 (0–3)	7 (4–9)	14 (7–20)	<0.01	4 (0–9)	4 (1–9)	3 (1–6)	6 (4–8)	0.51
Chronic bronchitis, emphysema	3 (2–5)	4 (2–6)	0.83	0 (0–0)	1 (0–3)	2 (0–4)	4 (2–6)	7 (2–11)	0.07	7 (1–12)	0 (0–0)	2 (0–4)	4 (2–6)	0.09
Other mental problem	2 (1–4)	3 (1–5)	0.48	16 (2–30)	6 (1–10)	3 (1–6)	1 (0–2)	0 (0–0)	<0.01	1 (0–3)	4 (1–9)	3 (0–5)	3 (2–4)	0.76
Substance abuse or addiction problem	2 (1–3)	1 (0–2)	0.29	0 (0–0)	3 (0–6)	4 (1–6)	1 (0–1)	0 (0–0)	0.01	1 (0–3)	2 (0–5)	2 (0-4)	2 (1–3)	0.98
Coronary thrombosis or heart attack	2 (0–3)	0 (0–1)	0.05	0 (0–0)	0 (0–0)	1 (0–2)	2 (0–3)	3 (0–5)	0.39	0 (0–0)	2 (0–5)	1 (0–2)	2 (1–3)	0.37
Stroke	1 (0–1)	1 (0–3)	0.21	0 (0–0)	0 (0–0)	0 (0–0)	2 (0–3)	1 (0–3)	0.22	1 (0–3)	0 (0–0)	2 (0–4)	1 (0–1)	0.46

Many comorbidities varied according to general characteristics among the population with SCI (Table 2b). There were more reports of back problems and asthma in females than in males. The incidences of high blood pressure/hypotension, high cholesterol, back problems, coronary heart disease or angina pectoris, and diabetes were lowest among the youngest participants and increased in every age group, being highest among persons aged ≥76. Depression and substance abuse or addiction problems were most common in the 46–60 age group with a statistically significant difference in those aged 61–75 years. Other mental problems were more frequent in the youngest age group than in the three oldest groups.

Between severity of SCI groups there were statistically significant differences in high cholesterol, high blood pressure/hypertension, and back problems (Table 2b), but the differences in high cholesterol vanished in the pairwise analyses after Bonferroni correction. High blood pressure/hypertension was more frequent in group T1–S5 AIS A, B, and C, as well as in group AIS D, than in group C5–8 AIS A, B, and C. Back problems were most frequent in group AIS D in comparison to all of the other groups. Additionally, there were some differences in the prevalence of the comorbidities from the aspect of time since injury and aetilogy (Supplementary Table C).

### Secondary health conditions

**Fig. 2:**
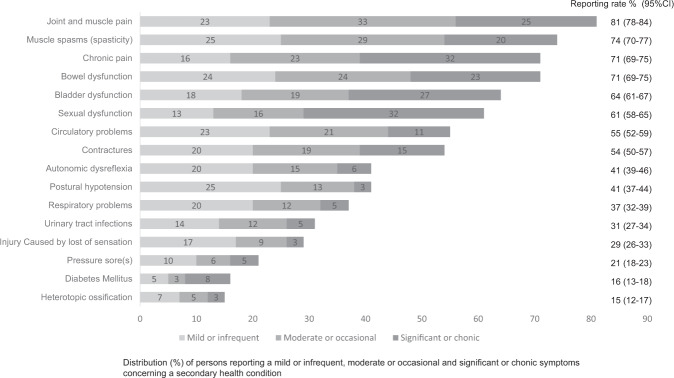
Prevalence of secondary health conditions (percentage with 95% confidence interval) in the Finnish Spinal Cord Injury study (884 participants).

Over 70% of the Finnish population with SCI reported suffering at least mildly or infrequently from joint and muscle pain, muscle spasms (spasticity), chronic pain, and bowel dysfunction (Fig. 2). Twelve participants (1%) reported no SHCs.

Males reported more sexual dysfunction, circulatory problems, and pressure sores than females. Bladder and bowel dysfunction, as well as joint and muscle pain were more frequent among females (Table 3). As in the comorbidities, several SHCs, such as heterotopic ossification, diabetes mellitus (included in the outcome of SHCs), respiratory problems, and contractures increased with age. Circulatory problems and autonomic dysreflexia differed between the age groups, but differences were not statistically significant after Bonferroni correction. Injuries due to loss of sensation, and chronic pain were highest among the 46–60 age group, with a statistically significant difference compared to the two oldest age groups (loss of sensation) and the 31–45 age group (chronic pain).

**Table 3:** Relative frequency (percentage with 95% confidence interval) of secondary health conditions in the Finnish Spinal Cord Injury study (884 participants) stratified by gender, age groups, and severity of SCI.

Secondary health condition	Gender		Age groups					Severity of SCI						
	Male	Female	*p*	20–30	31–45	46–60	61–75	≥ 76	*p*	C1-4 AIS A, B, and C	C5-8 AIS A, B, and C	T1-S5 AIS A, B, and C	AIS D at any injury level	*p*
	% (95%CI)	% (95%CI)		% (95% CI)	% (95% CI)	% (95% CI)	% (95% CI)	% (95% CI)		% (95% CI)	% (95% CI)	% (95% CI)	% (95% CI)	
Joint and muscle pain	79 (76–83)	84 (80–89)	0.03	79 (64–94)	78 (70–87)	83 (78–88)	82 (78–86)	76 (68–85)	0.48	77 (68–87)	84 (73–94)	85 (80–91)	80 (76–83)	0.48
Muscle spasms (spasticity)	75 (72–79)	70 (65–76)	0.12	73 (57–89)	73 (65–82)	75 (70–81)	74 (70–79)	69 (61–78)	0.82	87 (80–94)	80 (69–91)	67 (60–74)	73 (69–77)	<0.01
Chronic pain	70 (66–74)	73(68–79)	0.16	64 (46–81)	63 (53–72)	78 (73–84)	70 (65–75)	68 (58–77)	<0.01	68 (58–78)	67 (54–81)	68 (61–75)	73 (69–77)	0.61
Bowel dysfunction	69 (65-73)	77 (72–82)	0.01	73 (57–89)	77 (69–86)	73 (67–79)	70 (65–75)	68 (58–77)	0.54	71 (62–81)	80 (68–91)	84 (78–90)	67 (63–71)	<0.01
Bladder dysfunction	60 (55–64)	72 (67–78)	<0.01	64 (46–81)	70 (61–79)	60 (53–66)	64 (59–69)	69 (59–78)	0.23	68 (58–78)	65 (51–79)	73 (66–80)	60 (56–64)	0.03
Sexual dysfunction	70 (66–74)	45 (39–51)	<0.01	55 (37–72)	69 (59–78)	66 (59–72)	60 (55–65)	53 (42–63)	0.07	69 (59–79)	61 (47–75)	70 (63–77)	58 (53–62)	<0.01
Circulatory problems	53 (49–58)	60 (55–66)	0.03	39 (22–57)	47 (37–57)	57 (51–64)	57 (52–63)	61 (51–71)	0.03	60 (49–70)	65 (51–79)	68 (61–75)	50 (46–54)	<0.01
Contractures	56 (52–60)	48 (42–54)	0.08	24 (9–40)	40 (31–50)	53 (46–59)	58 (53–63)	61 (51–71)	<0.01	61 (51–73)	45 (30–59)	47 (39–55)	54 (50–59)	0.04
Autonomic dysreflexia	44 (40–48)	38 (32–44)	0.12	48 (30–66)	48 (38–58)	48 (41–55)	40 (35–45)	27 (18–36)	<0.01	63 (53–74)	63 (49–77)	43 (35–50)	36 (32–40)	<0.01
Postural hypotension	39 (37–49)	43 (37–49)	0.05	33 (16–50)	42 (32–52)	38 (31–44)	39 (34–45)	52 (41–62)	0.12	60 (49–70)	49 (34–63)	32 (25–40)	39 (35–43)	<0.01
Respiratory problems	36 (32–40)	36 (30–41)	0.49	21 (6–36)	26 (18–35)	33 (26–39)	39 (34–45)	46 (36–57)	<0.01	45 (34–56)	45 (30–59)	25 (18–32)	37 (33–41)	<0.01
Urinary tract infections	30 (26–34)	32 (26–37)	0.51	33 (16–50)	39 (30–49)	31 (25–37)	28 (24–33)	27 (18–36)	0.39	55 (44–66)	51 (37–66)	43 (36–51)	20 (17–24)	<0.01
Injury caused by loss of sensation	31 (27–35)	27 (21–32)	0.22	30 (14-47)	33 (24–43)	41 (35–48)	24 (19–28)	19 (11–28)	<0.01	31 (21–41)	27 (14-39)	30 (23-37)	30 (26–34)	0.98
Pressure sore(s)	24 (20–28)	14 (10–18)	<0.01	27 (11–43)	30 (21–39)	19 (14–24)	19 (15–23)	17 (9–25)	0.13	48 (37–59)	33 (19–46)	35 (27–42)	10 (7–13)	<0.01
Diabetes Mellitus	16 (12–19)	15 (11–20)	0.68	0 (0–0)	5 (1–9)	15 (10–19)	19 (15–24)	20 (12–29)	<0.01	14 (7–22)	18 (7–30)	12 (7–17)	17 (13–20)	0.66
Heterotopic ossification	15 (12–18)	14 (10–18)	0.94	0 (0–0)	12 (5–18)	12 (8–17)	17 (13–21)	22 (13–30)	0.01	18 (9–26)	10 (1–19)	18 (12–24)	14 (11–17)	0.34

Autonomic dysreflexia was more common in groups C1–4 AIS A, B, and C and C5–8 AIS A, B, and C than in the two other groups (Table 3). Postural hypotension was most common in group C1–4 AIS A, B and C with a statistically significant difference in comparison to groups T1-S5 AIS A, B, C, and AIS D. Group T1-S5 AIS A, B, C had fewer respiratory problems than other groups. Spasticity was the most common in group C1–4 AIS A, B, and C, and the difference was statistically significant in comparison to groups T1–S5 AIS A, B, and C and AIS D. Sexual dysfunction, circulatory problems as well as bowel and bladder dysfunctions were the most common in group T1–S5 AIS A, B and C, and the differences were statistically significant compared to group AIS D. Group AIS D had fewer pressure sores and urinary tract infections than all of the other SCI severity groups. Contractures differed in the severity of SCI groups, but this difference was not statistically significant in the pairwise comparisons after Bonferroni correction (Table 3). Additionally, there were some differences in the prevalence of the SHCs with regard to time since injury and aetilogy (Supplementary Table D).

### Multimorbidity

The mean number of reported comorbidities varied between the general and lesion characteristics (Table 4). The mean was lowest in the 20–30 years age group (0.48) and was highest among the participants aged 76 years or older (2.0). The scale range for the comorbidities was 0–12. For the SHCs, the lowest mean was also observed among the youngest participants (6.4), but the highest mean was observed in the SCI severity group C1-4 AIS A, B, and C (8.9). The scale range for the SHCs was 0–16. Statistically significant differences were found in the comorbidities between the age groups, in which the expected number increased with age (being 338% higher in the oldest age group than in the youngest). Persons with NTSCI had a 27% higher expected number of comorbidities than persons with TSCI in the adjusted model (Table 4).

**Table 4:** Incidence rate ratios (IRR) of multimorbidity for patient reported comorbidities (sum-score with scale-range 0–12) and secondary health conditions (sum-score with scale-range 0–16), in the Finnish Spinal Cord Injury study (884 participants), in relation to demographic and lesion characteristics.

		Comorbidities					Secondary health conditions				
Parameter	*n*	Mean (95% CI)	Unadjusted IRR (95% CI)	*p*	Adjusted IRR (95% CI)	*p*	Mean (95% CI)	Unadjusted IRR (95% CI)	*p*	Adjusted IRR (95% CI)	*p*
Gender											
Male	504	1.26 (1.14–1.38)	1		1		7.66 (7.37–7.95)	1		1	
Female	270	1.52 (1.35–1.70)	1.15 (0.96–1.38)	0.14	1.08 (0.90–1.31)	0.41	7.51 (7.14–7.88)	0.97 (0.83–1.14)	0.75	0.97 (0.84–1.17)	0.97
Age in years											
20–90 years					1.02 (1.02–1.03)	<0.01				1.00 (0.99–1.01)	0.44
Age groups											
20–30	31	0.48 (0.22–0.75)	1				6.39 (5.22–7.56)	1			
31–45	100	0.65 (0.45–0.85)	1.33 (0.67–2.64)	0.42			7.58 (6.95–8.21)	1.14 (0.75–1.73)	0.55		
46–60	214	1.30 (1.11–1.50)	2.68 (1.41–5.01)	<0.01			7.86 (7.39–8.32)	1.78 (0.80–1.74)	0.42		
61-75	336	1.49 (1.34–1.64)	3.23 (1.72–6.06)	<0.01			7.60 (7.25–7.94)	1.15 (0.78–1.69)	0.48		
≥ 76	93	2.01 (1.70–2.33)	4.38 (2.28–8.42)	<0.01			7.54 (6.87–8.21)	1.14 (0.74–1.74)	0.56		
Severity of SCI											
AIS D at any injury level	483	1.52 (1.39–1.65)	1		1		7.17 (6.87–7.47)	1		1	
T1-S5 AIS A, B, and C	162	1.21 (1.00–1.42)	0.91 (0.73–1.15)	0.44	1.04 (0.81–1.35)	0.74	8.02 (7.58–8.46)	1.13 (0.94–1.37)	0.20	1.12 (0.91–1.37)	0.31
C5-8 AIS A, B, and C	49	0.71 (0.45–0.98)	0.64 (0.42–0.96)	0.03	0.76 (0.49–1.16)	0.20	8.45 (7.40–9.50)	1.20 (0.88–1.65)	0.25	1.18 (0.85–1.65)	0.32
C1-4 AIS A, B, and C	80	1.01 (0.72–1.31)	0.75 (0.55–1.01)	0.06	0.88 (0.64-1.22)	0.44	8.93 (8.27–9.58)	1.27 (0.99–1.62)	0.06	1.26 (0.96–1.65)	0.09
Aetiology											
Traumatic	436	1.11 (1.99–1.23)	1		1		7.68 (7.37–7.99)	1		1	
Non-Traumatic	338	1.66 (1.50–1.83)	1.36 (1.14–1.63)	<0.01	1.27 (1.05–1.55)	0.01	7.51 (7.17–7.86)	0.97 (0.83–1.13)	0.69	1.01 (0.89–1.23)	0.60
Time since Injury											
1–5 years	311	1.57 (1.40–1.74)	1		1		7.19 (6.80–7.58)	1		1	
6–10 years	197	1.35 (1.15–1.54)	0.84 (0.68–1.05)	0.13	0.87 (0.69–1.08)	0.21	7.52 (7.07–7.96)	1.05 (0.87–1.27)	0.60	1.03 (0.85–1.25)	0.74
11–15 years	110	1.15 (0.90–1.41)	0.79 (0.60–1.04)	0.09	0.80 (0.60–1.07)	0.14	7.99 (7.39–8.6)	1.11 (0.88–1.40)	0.38	1.07 (0.84–1.25)	0.59
≥16 years	156	1.08 (0.89–1.26)	0.77 (0.60–1.05)	0.35	0.87 (0.66–1.15)	0.34	8.29 (7.83–8.76)	1.16 (0.94–1.42)	0.16	1.07 (0.85–1.35)	0.54

Unadjusted and adjusted incidence rate ratios (IRR) are from univariable and multivariable regression modelling using the negative binominal distribution and Wald tests for significance testing. Multivariable models are adjusted for all the variables mentioned in the column representing the adjusted incidence rate ratios.

## Discussion

This study assessed the comorbidities and SHCs among the Finnish population with SCI. Analyses of the comorbidities suggested that high blood pressure/hypertension and back problems were more common in the population with SCI than among the reference population, as was suspected from previous studies [3, 4]. However, CHD or angina pectoris was more frequent in the male reference population than in the male population with SCI. This result is contradictory in comparison to international studies which indicate that individuals with SCI are known to have a higher risk for cardiovascular diseases [22, 23]. Since it is also known that cardiovascular disease is one of the general chronic conditions among the Finnish population [24], our result raises a question of whether CHD is underdiagnosed among males with SCI in Finland. Thus, more attention should be paid to the possibility of this disease.

Ageing was a prominent factor in elevating the relative risk of multimorbidity in the analyses of the comorbidities among the population with SCI in Finland. Similar results have been reported in other studies [4, 25], and this can be considered as a natural phenomenon since ageing leads to an increase in morbidities [26]. This study suggests that the time since injury (i.e., ageing with SCI) is not associated with multimorbidity and that elderly persons with SCI often have other comorbidities as well. In Finland, the incidence of SCI is at 92 per million inhabitants annually; ~60% of these are NTSCI, and their mean age is 62 years [11, 27].

As expected, SHCs following SCI were often experienced by persons with cervical lesions classified by ISNCSCI as AIS A, B, and C. Higher injury level and more complete injury are related to a higher rate of medical complications [4–6, 8]. However, in the analyses of the relative risk of multimorbidity, there were no statistically significant differences between the severity of SCI groups. This result is contradictory, as Brinkhof et al. [5] indicated that persons with complete tetraplegia have a higher expected number for multimorbidity, as measured by a modified SCI-SCS. In their study, also persons with NTSCI had a higher relative risk for SHCs compared to persons with TSCI, which is different from our result. Regardless, the aetiology was a relevant indicator of multimorbidity in the analyses of the comorbidities in this study, since persons with NTSCI had a higher expected number of comorbidities than persons with TSCI.

As a single symptom, pain is one of the top three most frequent SHCs in several countries [5, 8, 28, 29], including Finland. In the present study, pain (chronic or joint/muscle) was experienced equally among all the characteristics. The negative influences that are associated with pain, such as depressive symptoms, more restricted and less satisfying participation, and low quality of life, can affect persons with SCI [28]. Unfortunately, number of persons with SCI who report pain problems has remained almost the same over the last decades [30].

### Strengths and limitations

As a benefit to the FinSCI study, from the planning stage to the realisation of this study, we have observed the active participation of persons with SCI with assistance from The Finnish Association of Spinal Cord Injured Akson. We believe that this has increased the response rate. Another advantage of this study was the access to the registered SCI outpatient clinic data, which made it possible to identify the majority of persons with SCI in Finland, and to reliably combine their general and lesion characteristics. However, the fact that the ISNCSCI classifications were completed by several physicians and physiotherapists between 2000 and 2018 has to be recognised. Even so, the protocol of the ISNCSCI was followed, and the latest classification from the medical records was used.

This study had several limitations. For example, a portion of the data were skewed; thus the results must be interpreted with caution. The high incidence of individuals in the SCI severity group AIS D was expected, since approximately 65% of the population with SCI in Finland belong to this group [11]. Group AIS D differed significantly from the other SCI severity groups because there were more elderly and non-traumatically injured persons, and most of the participants were injured within the last five years. Additionally, the younger population with SCI did not answer the survey as actively as the older individuals. The age and gender structure of the target population for FinSote and FinSCI are different and this aspect needs to be noted when interpreting the results. The national reference data were available for only four comorbidities, which makes these analyses incomplete. The data were collected by self-reported outcome measures, and the veracity of the comorbidities that were diagnosed or treated by a physician was not checked in the hospital records, which may have caused some inaccuracy in the data.

## Conclusions and future directions

The prevalence of comorbidities and SHCs was common among elderly persons with SCI. We found that the strongest predictor for multimorbidity was age. This finding emphasises the importance of geriatric-related knowledge in SCI because the number of senior citizens, the life expectancy of persons with SCI, and the incidence of SCIs will increase in the future [10, 11]. This can then cause a greater burden on Finland’s health care system, and it is necessary to create sufficient healthcare services with expertise in SCI. Additionally, possible preventive actions to minimise the risks of injuries should be evaluated early enough.

Persons with the most severe disabilities (that is, SCI severity groups C1–4 AIS A, B and C and C5–8 AIS A, B, and C) also reported a high prevalence of comorbidities and SHCs, even though there was no statistically significant difference between the severity of SCI groups. Additionally, persons with NTSCI had a higher risk of the multimorbidity than those with TSCI in the analyses of the comorbidities. Thus, we believe that the needs of persons with cervical lesions AIS A, B, or C, and persons with NTSCI should also receive more attention in future SCI research.

We found that the prevalence of SHCs in the Finnish population with SCI was high and that pain (as a single symptom) was consistently one of the main problems in their daily lives. Therefore, the regular evaluation of SHCs, especially pain, and the effective use of pharmacological and nonpharmacological methods to ease SHCs are extremely important.

This study suggests that in Finland, the population with SCI has more comorbidities than the reference population. Further research is needed to assess this issue in more detail.

## Supplementary information

Supplement

## Data Availability

The authors will consider any reasonable requests to access the data.
