# Supplementary material for: Prevalence of comorbidities and secondary health conditions among the Finnish population with spinal cord injury
Source: Spinal Cord. 2021 Sep 11;60(7):618–27. doi: 10.1038/s41393-021-00704-7 (PMC9287167; doi:10.1038/s41393-021-00704-7)
Supplement: Supplementary file 1 — Supplement [file 41393_2021_704_MOESM1_ESM.pdf]

Susanna Tallqvist, MSc; Anna-Maija Kauppila MD, PhD; Aki Vainionpää, MD, PhD; Eerika Koskinen, MD, PhD; Paula Bergman, MSc; Heidi Anttila, PhD; Harri Hämäläinen, MD, PhD; Anni Täckman, BBA; Mauri Kallinen, MD, PhD; Jari Arokoski, MD, PhD; and Sinikka Hiekkala, PhD

Prevalence of comorbidities and secondary health conditions among the Finnish population with spinal cord injury

Spinal Cord, 2021

Legends:

Supplementary Figure A. Locations of the three SCI outpatient clinics.

Supplementary Table A. Items used in the analyses of prevalence of comorbidities and secondary health conditions in the Finnish Spinal Cord Injury Study

Supplementary Table B. Internal analyses of the participants in the Finnish Spinal Cord Injury study (884 participants).

Supplementary Table C. Relative frequency (percentage with 95% confidence interval) of comorbidities diagnosed or treated by a physician in the Finnish Spinal Cord Injury study (884 participants), stratified by time since injury and aetiology.

Supplementary Table D. Relative frequency (percentage with 95% confidence interval) of secondary health conditions in the Finnish Spinal Cord Injury study (884 participants), stratified by time since injury and aetiology.

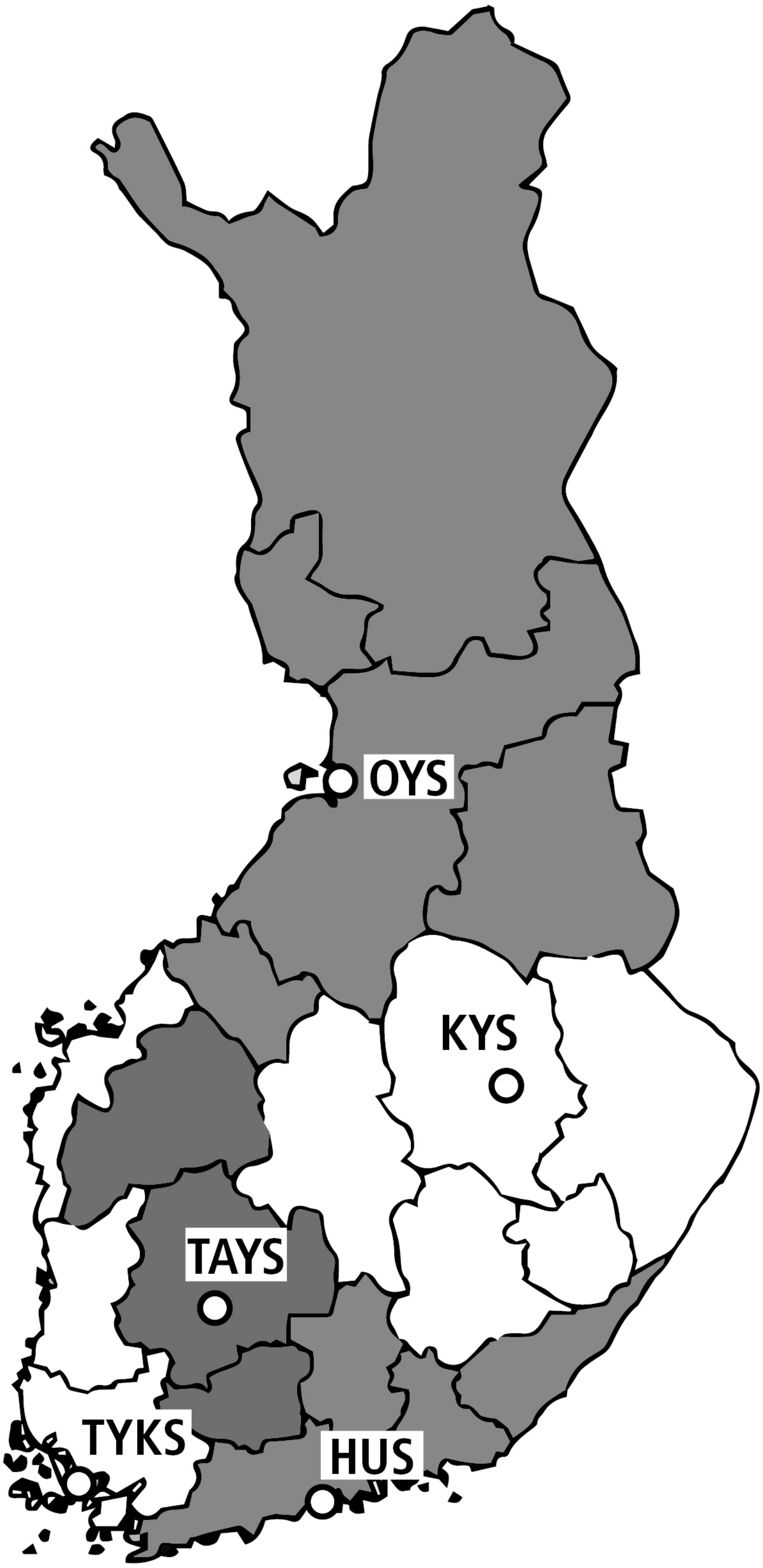

Supplementary Figure A. Locations of the three SCI outpatient clinics. There are 5 university hospitals in Finland. According to the Health Care Act and government decree, acute care, immediate rehabilitation and life-long multi-professional follow-up, care and rehabilitation of persons with spinal cord injury (SCI) have been centralized at 3 university hospitals: Oulu (OYS), Tampere (TAYS) and Helsinki (HUS) University Hospitals. These hospitals serve the whole SCI population in Finland. Turku (TYKS) and Kuopio university hospital (KYS) send their persons with SCI from their hospital district areas mainly to TAYS, but depending from the resources of the services in SCI centers in OYS, TAYS, and HUS. Population in hospital district areas; OYS 0,74 milj., TAYS 0,90 milj., HUS 2,2 milj., TYKS 0,9 milj., KYS 0,80 milj.

Supplementary Table A. Items used in the analyses of prevalence of comorbidities and secondary health conditions in the Finnish Spinal Cord Injury Study		
Instrument	Instrument Label	ICF category
American Spinal Injury Association Impairment Scale (AIS)	Muscle power	b730, Muscle power functions*
Spinal cord Injury Secondary Conditions Scale (SCI-SCS)	1. Pressure sore(s)	b810, Protective functions of the skin*
	2. Injury caused by loss of sensation	b270, Sensory functions
	3. Muscle spasms (spasticity)	b735, Muscle tone functions*
	4. Contractures	b710, Mobile of joint functions*
	5. Heterotopic bone ossification	s7700, Bones
	6. Diabetes mellitus	Health condition
	7. Bladder dysfunction	b620, Urination functions*
	8. Bowel dysfunction	b525, Defecation functions *
	9. Urinary tract infections	Health condition
	10. Sexual dysfunction	b640, Sexual dysfunction*
	11. Autonomic dysreflexia	Health condition
	12. Postural hypotension	b420, Blood pressure functions
	13. Circulatory problems	b415, Blood vessel functions
	14. Respiratory problems	b440, Respiratory functions
	15. Chronic pain	b280, Sensation of pain*
	16. Joint and muscle pain	b2801, Pain in body part*
National Study of Health, Well-being, and Service (FinSote)	Have you had any of the following conditions diagnosed or treated by a physician during the last 12 months?: High blood pressure of hypertension; Stroke; High cholesterol; Coronary thrombosis or heart attack; Coronary heart disease or Angina pectoris; Back problems (spondylosis, sciatica or other spinal disease; Chronic bronchitis, emphysema; Depression; Other mental problem; Asthma; Diabetes; Substance abuse or addiction problem	Health condition**
* ICF category belongs to FinSCI Dataset; ** If the meaningful concept refer to a diagnosis or a health condition, the meaningful concept will be assigned health condition		

Supplementary Table B. Internal analyses of the participants in the Finnish Spinal						
		Severity of SCI				
		C1-4 AIS A, B, and C	C5-8 AIS A, B, and C	T1-S5 AIS A, B, and C	AIS D at any injury level	p value
Gender		n (%)	n (%)	n (%)	n (%)	<0.01
	Male	79 (83%)	43 (78%)	123 (67%)	332 (60%)	
	Female	16 (17%)	12 (22%)	61 (33%)	218 (40%)	
Age groups						< 0.01
	20-30	5 (5%)	5 (9%)	10 (5%)	14 (2%)	
	31-45	14 (15%)	13 (24%)	43 (23%)	47 (7%)	
	46-60	21 (22%)	12 (22%)	47 (26%)	158 (29%)	
	61-75	47 (50%)	20 (36%)	73 (40%)	246 (45%)	
	≥ 76	8 (8%)	5 (9%)	11 (6%)	94 (17%)	
Time since SCI						<0.01
	1-5 years	21 (22%)	10 (18%)	28 (15%)	294 (53%)	
	6-10 years	23 (24%)	9 (16%)	46 (25%)	149 (27%)	
	11-15 years	11 (12%)	14 (26%)	39 (21%)	64 (12%)	
	≥ 16	40 (42%)	22 (40%)	71 (39%)	43 (8%)	
Aetiology						<0.01
	Traumatic	83 (87%)	45 (82%)	127 (69%)	237 (43%)	
	Non traumatic	12 (13%)	10 (18%)	57 (31%)	313 (57%)	

Supplementary Table C. Relative frequency (percentage with 95% confidence interval) of comorbidities diagnosed or treated by a physician in the Finnish Spinal Cord Injury study (884 participants), stratified by time since injury and aetiology

Comorbidity	Time since SCI					Aetiology		
	1-5 years	6-10 years	11-15 years	≥ 16		Traumatic	Non traumatic	
	% (95%CI)	% (95%CI)	% (95%CI)	% (95%CI)	*p*	% (95%CI)	% (95%CI)	*p*
High blood pressure or hypertension	45 (39-50)	37 (31-43)	30 (22-39)	33 (26-40)	0.01	31 (27-35)	47 (42-52)	<0.01
Back problems (spondylosis, sciatica or other spinal disease)	32 (27-37)	28 (22-34)	26 (18-33)	22 (16-29)	0.14	21 (18-25)	36 (32-41)	<0.01
High cholesterol	24 (20-29)	22 (17-28)	17 (10-23)	20 (14-26)	0.34	20 (17-24)	24 (19-28)	0.26
Diabetes	20 (15-24)	18 (13-23)	14 (8-20)	14 (9-20)	0.30	14 (11-17)	21 (17-25)	0.01
Depression	16 (12-20)	9 (6-13)	11 (6-17)	13 (8-18)	0.14	11 (8-14)	16 (12-19)	0.04
Asthma	11 (8-15)	8 (4-12)	11 (6-17)	8 (4-12)	0.47	8 (6-11)	11 (8-15)	0.14
Coronary heart disease or Angina pectoris	5 (3-8)	5 (2-8)	6 (2-10)	3 (1-6)	0.76	4 (2-5)	7 (4-9)	0.04
Chronic bronchitis, emphysema	4 (2-6)	4 (1-6)	2 (0-5)	2 (0-5)	0.59	4 (2-5)	3 (1-5)	0.64
Other mental problem	4 (2-6)	3 (1-5)	3 (0-6)	1 (0-2)	0.22	3 (1-4)	3 (1-5)	0.87
Substance abuse - or other addiction problem	1 (0-3)	2 (0-4)	1 (0-2)	2 (0-4)	0.77	2 (1-3)	1 (0-2)	0.24
Coronary thrombosis or heart attack	1 (1-2)	2 (0-4)	0 (0-0)	2 (0-4)	0.36	0 (0-0)	3 (1-4)	0.01
Stroke	1 (0-2)	0 (0-0)	1 (0-2)	2 (0-4)	0.30	1 (0-1)	1 (0-2)	0.49

Supplementary Table D. Relative frequency (percentage with 95% confidence interval) of secondary health conditions in the Finnish Spinal Cord Injury study (884 participants), stratified by time since injury and aetiology								
Secondary health condition	Time since SCI					Aetiology		
	1-5 years	6-10 years	11-15 years	≥ 16	*p*	Traumatic	Non traumatic	p
	% (95%CI)	% (95%CI)	% (95%CI)	% (95%CI)				
Joint and muscle pain	79 (75-84)	78 (72-81)	84 (77-91)	86 (81-92)	0.80	79 (76-83)	83 (79-87)	0.53
Chronic pain	71 (66-76)	74 (68-80)	72 (64-81)	66 (59-74)	0.60	69 (64-73)	74 (69-79)	0.11
Bowel dysfunction	64 (59-69)	71 (65-77)	80 (73-88)	82 (76-88)	<0.01	73 (68-77)	70 (77-75)	0.33
Bladder dysfunction	56 (51-62)	71 (65-77)	65 (56-74)	70 (62-77)	0.01	62 (58-67)	66 (61-71)	0.28
Sexual dysfunction	58 (53-64)	63 (56-69)	62 (53-71)	67 (60-74)	0.31	64 (60-69)	29 (53-64)	0.10
Circulatory problems	50 (44-55)	58 (51-65)	60 (51-70)	63 (55-70)	0.04	54 (49-59)	58 (53-63)	0.26
Contractures	54 (48-59)	50 (43-57)	53 (44-54)	56 (49-64)	0.60	52 (48-57)	54 (49-59)	0.41
Muscle spasms (spasticity)	54 (48-59)	50 (43-57)	53 (44-63)	56 (49-64)	0.60	52 (48-57)	54 (49-59)	0.41
Autonomic dysreflexia	38 (32-43)	38 (32-45)	44 (35-54)	54 (47-62)	<0.01	47 (42-52)	36 (31-41)	<0.01
Postural hypotension	41 (35-46)	42 (35-49)	33 (24-42)	42 835-50)	0.27	40 (35-45)	41 (36-46)	0.95
Respiratory problems	40 (34-45)	35 (28-41)	35 (26-44)	30 (23-37)	0.47	36 (32-41)	35 (30-40)	0.91
Urinary tract infections	25 (20-29)	27 (21-34)	37 (28-46)	42 (35-50)	<0.01	35 (30-39)	25 (21-30)	<0.01
Injury caused by loss of sensation	30 (25-35)	25 (19-31)	39 (30-48)	27 (20-34)	0.13	29 (24-33)	31 (26-36)	0.65
Pressure sore(s)	11 (8-15)	21 (16-27)	28 (19-36)	34 (26-41)	<0.01	25 (21-29)	14 (11-18)	<0.01
Diabetes Mellitus	18 (14-22)	17 (12-22)	10 (4-16)	13 (7-18)	0.30	12 (9-15)	20 (15-24)	0.01
Heterotopic ossification	15 (11-18)	13 (8-18)	15 (9-22)	18 (12-24)	0.80	16 (12-19)	14 (10-18)	0.71
